# COVID-19 outcomes in individuals with severe alpha-1 antitrypsin deficiency in Sweden

**DOI:** 10.1038/s41598-026-35016-4

**Published:** 2026-01-12

**Authors:** Suneela Zaigham, Eeva Piitulainen, Hanan Tanash

**Affiliations:** Department of Respiratory Medicine, Lund University, Skåne University Hospital, Tora Kjellgrensgatan 17, Plan 4, S-205 02 Malmö, Sweden

**Keywords:** COVID, Alpha-1 antitrypsin deficiency, Diseases, Health care, Medical research, Risk factors

## Abstract

We have previously found using questionnaire/interview data on COVID-19 outcomes, that most subjects with severe alpha-1-antitrypsin deficiency (AATD) exhibit mild COVID-19 infection and those who additionally have COPD are at increased risk of severe COVID-19. We used objective information on COVID-19 outcomes in severe AATD (PiZZ) from the Swedish population and compared the risk of severe COVID-19 in severe AATD to the risk of severe COVID-19 in the general population. We cross-linked the Swedish National AATD Registry with the Swedish National Patient Register to identify subjects with severe AATD that required COVID-19-related hospitalization between March 2020 until June 2023. Standardised incidence ratio (SIR) was calculated using observed COVID-19 hospitalisations in severe AATD and expected COVID-19 hospitalisations from the general population. Logistic regression was used to calculate odds of moderate or severe COVID-19 in severe AATD in relation to pre-existing comorbidities. In 1228 subjects with severe AATD, there were 61 cases of COVID-19-related hospitalisations (severe COVID-19) and 32 moderate COVID-19 during follow-up. The observed hospitalizations for COVID-19 in subjects with severe AATD exceeded that expected in the general population by over threefold (SIR = 3.4). Odds of COVID-19 was elevated in subjects with severe AATD with COPD or CVD (OR 2.70 (1.52–4.80) and 3.51 (2.04–6.06), respectively. The majority of subjects with severe AAT deficiency had mild COVID-19 during the pandemic. Of subjects that did have severe COVID-19, pre-existing co-morbidities were common, potentially explaining the higher rate of COVID-19 hospitalisations in severe AATD relative to the general population.

## Introduction

In December 2019, the coronavirus disease (COVID-19), caused by severe acute respiratory syndrome coronavirus 2 (SARS-CoV-2), emerged in China and rapidly escalated into a global pandemic. The clinical responses to COVID-19 varied significantly, ranging from asymptomatic or mild presentations to severe illness and death, across different populations and regions. Non-genetic factors, such as age, comorbidities (e.g., cancer and cardiovascular diseases), and environmental exposures (e.g., air pollution), have been implicated in differential susceptibility to SARS-CoV-2 infection^1–3^. Additionally, host genetic factors may play a role in modulating disease severity^4^. Understanding the interplay between non-genetic and genetic influences on host immune function is essential for unravelling why some individuals develop severe disease and others experience mild or asymptomatic infections.

Among genetic factors, one candidate gene might be SERPINA1 which encodes the alpha-1-antitrypsin (AAT) protein. AAT is an acute-phase protein that is mainly produced. by liver cells and subsequently secreted into the plasma. In addition to its anti-proteinase function and inhibition of serine proteinases including neutrophil elastase, cathepsin G, and proteinase 3, AAT has several known non-proteinase effects including anti-inflammatory and immunomodulatory characteristics and antimicrobial/antiviral properties^5,6^. Severe hereditary AAT deficiency is characterized by decreased serum level and abnormal AAT function, and it is also associated with an increased risk of liver and lung diseases.

The transmembrane serine protease 2 (TMPRSS2) is crucial for SARS-CoV-2 cell entry and is inhibited by AAT^5–7^. It has therefore been suggested that AAT can inhibit extracellular proteolytic activity and limit SARS-CoV-2 cell–cell spread and dissemination^7^. In AAT deficiency, serine protease levels are not suppressed allowing for high amounts to be available for the virus^8^. Low AAT levels have therefore been found to be a predictive factor associated with high likelihood of severe COVID-19 illness^9^.

Despite the implications for global health, the inflammatory characteristics of patients with COVID-19 are incompletely understood, as are the inflammatory mediators and attendant molecular mechanisms underlying them. Hence, genetic variations may be among factors that contribute to differences in COVID-19 infection rates and severity^10^. Both severe COVID-19 outcomes and AAT deficiency has had a high prevalence in several European countries^8,11,12^. During the pandemic, countries with a high prevalence of AAT deficiency had a sustained high level of COVID-19 cases and deaths in the initial 6 months whereas in countries with a low prevalence of AAT deficiency there appeared to be an attenuated variant of COVID-19 with a decreasing case-fatality rate^8^. Some studies have also suggested that patients with AAT deficiency should have a higher risk of developing severe COVID-19 outcomes^9,13–15^. We have previously used the Swedish AATD register to assess COVID-19 outcomes in these subjects with severe AAT deficiency (phenotype PiZZ) and found that most subjects with severe AAT deficiency had mild flu-like symptoms of COVID-19 during the pandemic and patients who also had COPD were at increased risk of severe COVID-19 infection^16^. However, this study was based on interview/questionnaire responses and therefore was a subjective overview of the pandemic in severe AAT deficiency^16^.

We aimed to link the Swedish AATD register with the Swedish National Patient Register to gain a comprehensive objective understanding of the COVID-19 pandemic in subjects with severe AAT deficiency (PiZZ) in Sweden. We also aimed to compare the risk of severe COVID-19 outcomes in these subjects with severe COVID-19 outcomes in the general population and to assess the role of pre-existing comorbidities in COVID-19 outcomes.

## Methods

### Study population

The individuals with severe AATD (PiZZ) included in this study were selected from the Swedish National AATD Register, which is described in detail elsewhere^17^. The register was established in 1991 and inclusion criteria are: 1) age ⩾18 years, 2) a diagnosis of severe AATD (phenotypes PiZZ and PiZNull verified by isoelectric focusing) and 3) written, informed consent to participate.

Briefly, the results from the individual’s clinical examination, results of the laboratory analyses of liver function and lung function tests measured by means of spirometry, performed at the individual’s local hospital or home clinic every two years, are reported to the AATD Register by the attending physician, via a questionnaire. The questionnaire also includes questions on medical diagnoses, smoking habits and tobacco consumption.

### COVID-19 outcomes

Severe COVID-19 infection was defined as a hospitalization due to COVID-19 infection leading to an inpatient stay. Moderate COVID-19 infection was defined as seeking care for COVID-19 at the emergency department or outpatient clinic. The control group consisted of individuals with no documented COVID-19 infection or with only mild disease. Because COVID-19 status in this group was not verified in any registry, these individuals were assumed either to have remained uninfected or to have experienced a mild, self-limiting illness compatible with COVID-19 that was managed in the community.

Cross-linking the Swedish AATD Registry with the Swedish National Patient Register was conducted to identify patients with severe COVID-19 infections requiring hospitalization, as well as those with moderate COVID-19 infections who sought care at emergency departments or hospital outpatient clinics. Subjects with mild infection were not linked to any primary care register. To determine COVID-19 as the primary cause of death, the AATD Registry was also linked to the Swedish Causes of Death Register. Data on patient baseline characteristics, including smoking habits, method of identification, and spirometry results at the time of inclusion and the most recent follow-up, were obtained from the registry. The data on COVID-19 infection in the general population were obtained from the Swedish National Board of Health and Welfare^18^.

### Diagnoses

Data on vital status and causes of death were obtained from the Swedish National Register of Causes of Death up to September 1st, 2023, for all the individuals in the study, however for the purposes of the study we used follow-up time until 30^th^ June 2023 to be in line with the data available from the general population from the Swedish National Board of Health and Welfare. Data on diagnoses of COVID-19 infection and other conditions were obtained from the Swedish National Patient Register (SNPR). The SNPR covers more than 99% of all hospitalizations since 1987 and about 80% of all hospital-based outpatient care since 2001 nationwide, including at outpatient clinics for infectious disease^19^. Diagnoses were coded according to the 9th (before 1996) and 10th WHO International Classification of.

Disease (ICD) system; COVID-19 (U07.1, U08.9, U09.9, U10.9). ICD coding was also used to define subjects with cardiovascular diseases (CVD), COPD and diabetes for logistic regression models. (CVD: ICD-9; 390–459 and ICD-10; I00-I99, COPD: ICD-9; 490–492 and 496 and ICD-10; J40-J44 and diabetes ICD-9; 250.x and ICD-10 E10 and E11).

### Ethical considerations

The Swedish National AATD Registry is approved by the Regional Ethical Review Board, Lund, Sweden and by the Swedish Data Inspection Board. The study was carried out according to the principles of the Declaration of Helsinki.

All individuals included in the Swedish National AATD Registry had provided written informed consent at the time of inclusion, including consent for collection of data from medical records and national patient registers for research purposes. The present study represents a retrospective analysis of fully anonymised registry data conducted within the scope of this original consent. The present study was approved by the Regional Ethical Review Board, Lund, Sweden and Swedish Ethical Review Authority (2014/427and 2020–03,814).

### Statistical analyses

Data at inclusion were tabulated using frequencies and percentages for categorical variables. To compare categorical data, the *χ*^*2*^ -test was used. For continuous data, means with standard deviations (SD) were used. Comparisons of continuous variables were analysed using ANOVA. The follow-up time was from December 1^st^ 2019 to the date of death or study end on September 1^st^, 2023 however for the purposes of this study, follow-up between 1^st^ March 2020 and 30^th^ June 2023 was used to be in line with the records from the general population.

Hospitalisation rate for COVID-19 (per 100,000) in the general population of Sweden between 1^st^ March 2020 and 30^th^ June 2023 were used to calculate the standardised incidence ratio (SIR) for COVID-19 in the current cohort. The present cohort was divided into age-sex groups, and the number of COVID-19 hospitalisations and the number of person-years follow-up were calculated for each group. Age for age-sex groups was calculated for each subject for each year they were part of the study, so that their study time in person-years was taken into account within each age bracket of the age-sex groups. Hospitalisation rate for COVID-19 in the general population was multiplied by person-years for each age-sex group to get the expected hospitalizations for the present cohort.

Odds ratio (OR) for COVID-19 was calculated in subjects with severe AATD (PiZZ) in relation to pre-existing comorbidities. Both moderate (emergency visits and outpatient visits) and severe COVID-19 (hospitalisations) was used as a COVID-19 event and risk was calculated relative to mild or no COVID-19. Both unadjusted and adjusted (age at start of follow-up, sex and smoking) risks were calculated.

A two-sided p-value of less than 0.05 was considered statistically significant. Statistical analyses were performed with the Statistical Package for the Social Sciences (SPSS) version 27.0 (IBM Corporation, Armonk, NY, USA).

## Results

**Fig. 1:**
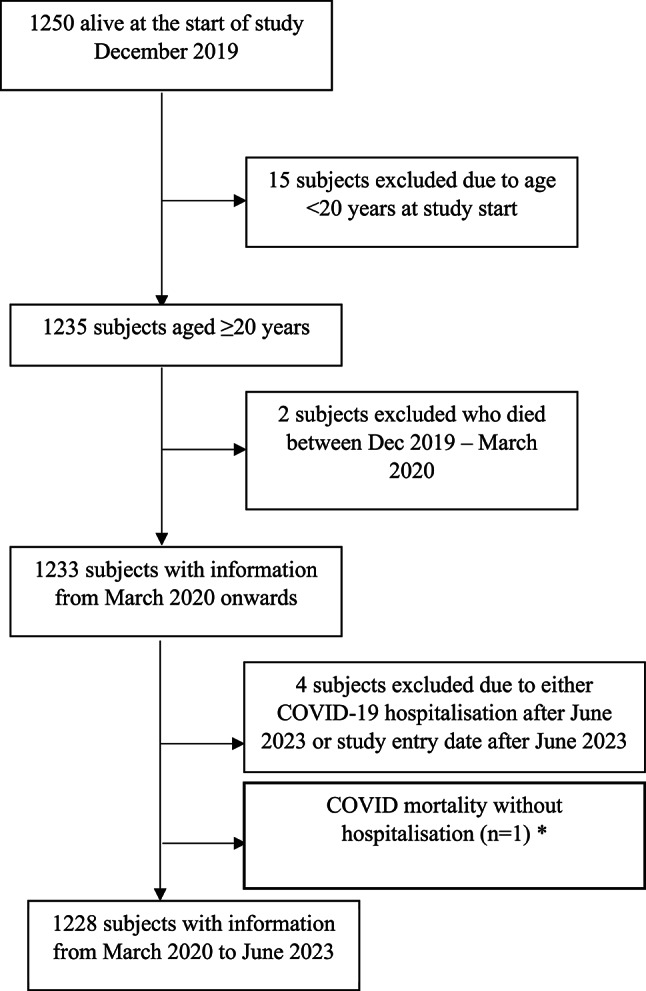
Flow of subjects through the study.

Of the 1250 subjects alive at the start of the original study (December 2019), 15 subjects were excluded due to age < 20 years at start of follow-up, (as no COVID-19 hospitalisations occurred in this age group) 2 subjects were excluded due to death between December 2019 and March 2020 (start of follow-up for present study). A further 4 subjects were excluded due to a COVID-19 hospitalization after the end of follow-up time (June 2023) or subject entered the study after the last day of follow-up for the present study. One subject died with COVID-19 as the underlying cause of death outside of the hospital, therefore they were excluded from the main analysis of severe COVID-19 hospitalisations but descriptive information on their death is included. Flow of subjects through the study is displayed in Fig. 1.

None of the individuals included in this study received any regular AAT augmentation therapy. Of the 1228 subjects in the cohort, there were 61 cases of COVID-19-related hospitalisations (severe COVID-19), 32 visits to either the emergency department or an outpatient clinic due to COVID-19 (moderate COVID-19) and 1135 cases of either mild COVID-19 or no COVID-19 infection during follow-up between March 2020 and June 2023.

Subjects who had severe COVID-19 were older (mean age 66.4 years *vs* 56.1 and 58.1 years for mild/no COVID-19 and moderate COVID-19, respectively, p < 0.001), had a higher proportion of ever-smokers (64% *vs* 41 and 28% for mild/no COVID-19 and moderate COVID-19, respectively, p < 0.001), had a higher number of pack-years, and had a higher proportion of subjects with other co-morbidities such as COPD, CVD, diabetes and lung transplantation compared to subjects with mild or no COVID-19 and moderate COVID-19 (all p-values < 0.001). They also had a lower mean FEV_1_ at study inclusion (2.48 (± 1.16), litres) and a lower FEV_1_/FVC ratio (0.58 (± 0.20) compared to subjects with mild/no COVID-19 or moderate COVID-19, (p-value < 0.001) (Table 1) and a larger proportion of them were on long-term oxygen therapy prior to COVID-19 infection (12% *vs* 2 and 3% for mild/no COVID-19 and moderate COVID-19, respectively, p < 0.001). 

The standardised incidence ratio (SIR) for COVID-19 hospitalizations in subjects with severe AAT deficiency is displayed in Table 2. Subjects with severe AAT deficiency had an SIR of 3.4, indicating the observed hospitalizations for COVID-19 in subjects with severe AAT deficiency exceeds that which is expected in the general population, by over threefold.

**Table 1:** Baseline characteristics of the study population (n = 1228).

	Mild or no COVID-19 (n = 1135)	Moderate COVID-19 (n = 32)	Severe COVID-19 (n = 61)	p-value
Age at start of follow-up (years)	56.1 (± 15.0)	58.1 (± 14.4)	66.4 (± 11.9)	< 0.001
Men, n (%)	530 (47)	13 (41)	34 (56)	0.280
Ever-smokers n, (%)	470 (41)	9 (28)	39 (64)	< 0.001
Pack-years*	10.3 (± 10.0)	13.8 (± 8.0)	18.4 (± 14.3)	< 0.001
COPD n, (%)	623 (55)	23 (72)	53 (87)	< 0.001
CVD n, (%)	381 (34)	15 (47)	49 (80)	< 0.001
Diabetes n, (%)	69 (6)	4 (13)	10 (16)	< 0.001
Asthma n, (%)	160 (14)	3 (9)	10 (16)	0.082
Lung transplantation n, (%)	31 (3)	2 (6)	12 (20)	< 0.001
Total deaths n, (%)	86 (8)	4 (13)	20 (33)	< 0.001
Death due to COVID-19 n, (%)	0	0	7 (12)	< 0.001
FEV_1_ at inclusion (litres) **	3.15 (± 1.25)	3.05 (± 1.25)	2.48 (± 1.16)	< 0.001
FEV_1_ (% predicted) at inclusion**	89 (± 26)	86 (± 27)	70 (± 28)	< 0.001
FEV_1_/VC at inclusion**	0.72 (± 0.18)	0.71 (± 0.15)	0.58 (± 0.20)	< 0.001
Long-term oxygen therapy n, (%)	22 (2)	1 (3)	7 (12)	< 0.001

Data is presented as mean (± SD) unless otherwise stated. Moderate COVID: Visit to emergency department or outpatient clinic. Severe COVID: Hospitalisation due to COVID. P-value: ANOVA test for linearity for continuous variables, and chi square test linear by linear association for categorical variables. * Pack-years information available in 458 ever-smokers. **FEV_1_ measured in 1141 subjects, FEV_1_%predicted in 1112 subjects and FEV_1_/VC measured in 1014 subjects. COPD: Chronic Obstructive Pulmonary Disease, CVD: Cardiovascular disease, FEV1: Forced expiratory volume in 1 s, VC: Vital capacity. Long-term oxygen therapy refers to subjects needing supplemental oxygen prior to COVID infection.

**Table 2:** Standardised incidence ratio (SIR) for COVID-19 hospitalisations.

Age-sex groups	Observed COVID-19 hospitalisations in severe AATD (2020–2023)	Number of person-years in study population (2020–2023)	COVID-19 hospitalisations in the general population (2020–2023)	Hospitalisation rate for COVID-19 (per 100.000) in general population (2020–2023)	Expected hospitalisations in study population
Men 20–49 yrs	2	536.85	10 727	155.71	0.84
Men 50–79 yrs	27	1107.48	38 939	679.1	7.52
Men 80–99 yrs	5	56.48	19 367	2472.82	1.40
Women 20–49 yrs	4	622.77	11 668	71.80	0.45
Women 50–79 yrs	23	1243.85	25 458	438.92	5.46
Women 80–99 yrs	0	136.74	18 339	1653.80	2.26
**TOTAL**	**61**				**17.92**
**Standardised Incidence ratio = 3.4**					

AATD: Alpha-1 Antitrypsin deficiency.

Odds of moderate or severe COVID-19 in relation to the presence of co-morbidities in subjects with severe AAT deficiency is presented in Table 3. OR for COVID-19 was over 2.5-fold in presence of concurrent COPD (OR 2.70 (1.52–4.80) *vs* no COPD (reference) after adjustments for age, sex and smoking and was 3.5-fold higher in presence of concurrent CVD 3.51 (OR 2.04–6.06) *vs* no CVD (reference). A significant increase in risk of COVID-19 was also found in presence of diabetes (*vs* no diabetes) and previous lung transplantation (*vs* no transplantation) (OR 2.13 (1.14–3.99) and OR 5.25 (2.58–10.65) after adjustments, respectively). 

There were 8 (0.7%) COVID-19-related deaths during follow-up, seven of which occurred in participants with a documented COVID-19 hospitalisation and one death occurred outside of hospital/without a hospital admission. Mean age of death was 76 years (± 10.7). Seven out of eight subjects who died due to COVID-19 during follow-up also suffered from COPD and all subjects also had some form of CVD (including hypertension). Mean FEV_1_ (litres) in this group was 2.60 (± 0.88). No subjects who had a COVID-19-related death were on long term supplemental oxygen preceding COVID-19.

**Table 3:** Odds ratio for moderate or severe COVID-19 in subjects with severe AAT deficiency in relation to pre-existing comorbidities.

	Mild or no COVID-19 (reference) (n = 1135)	Moderate or severe COVID-19 (n = 93)	p-value
**COPD**			
Unadjusted	1.00	3.67 (2.14–6.30)	< 0.001
Adjusted	1.00	2.70 (1.52–4.80)	< 0.001
**CVD**			
Unadjusted	1.00	4.37 (2.77–6.89)	< 0.001
Adjusted	1.00	3.51 (2.04–6.06)	< 0.001
**Diabetes**			
Unadjusted	1.00	2.74 (1.48–5.08)	0.001
Adjusted	1.00	2.13 (1.14–3.99)	0.018
**Lung transplantation**			
Unadjusted	1.00	6.31 (3.23–12.35)	< 0.001
Adjusted	1.00	5.25 (2.59–10.67)	< 0.001

Adjusted for age at start of follow-up, sex, smoking. Mild COVID was defined as COVID managed in the community without contact with secondary healthcare. Moderate or severe COVID defined by visit to emergency department or outpatient clinic and COVID related hospitalisations. COPD; Chronic Obstructive Pulmonary Disease, CVD: Cardiovascular disease.

## Discussion

This large, register-based study of individuals with severe AAT deficiency shows that severe COVID-19 was relatively uncommon during the pandemic. The majority of patients either experienced mild infection managed in the community or remained uninfected, requiring no medical intervention. Among those who developed severe COVID-19, most had significant pre-existing comorbidities, particularly COPD and CVD, and were current or former smokers. These characteristics are well-recognized risk factors for severe COVID-19 and likely explain the higher hospitalization rates observed in this subgroup. Our findings suggest that severe AAT deficiency itself may not substantially increase the risk of severe COVID-19, but that outcomes are strongly influenced by co-existing health conditions and smoking history, mirroring patterns seen in the general population. This study adds to and supports our previous findings of COVID-19 infection in severe AAT deficiency^16^, now with objective register-based outcomes for COVID-19 infection to define severe COVID-19 infection during the pandemic. There have been some disparities in the findings from previous studies that have assessed if AAT deficiency was related to COVID-19 outcomes during the pandemic. Some previous studies have found that the presence of mutations associated with AAT deficiency and AAT levels below 116 mg/dL were some predictors of severe COVID-19 infection, suggesting that patients with AAT deficiency are at a higher risk of developing severe COVID-19 infection^9^. On the other hand, in the UK Biobank study it was found that subjects with mild AAT deficiency (PiMZ and PiMS) did not have an increased risk of COVID-19 infection^20^. As the number of subjects with severe AAT deficiency was low, no conclusions were made regarding the risk of COVID-19 in more severe AAT deficiency. Similarly, Nygren et al. did not find mild AAT deficiency to be a strong risk factor for COVID-19 infection or hospitalisation, however due to a small study size and no cases of moderate or severe AAT deficiency, they concluded that large registry-based studies are needed to adequately quantify this risk^21^.

European studies of AAT deficiency and COVID-19 during the pandemic have found a frequency of 3.8% for COVID-19 infection in a cohort of 209 subjects from the Italian registry of severe AAT deficiency, of which 78 subjects had the PiZZ genotype (37.3%), 53 had the PiSZ genotype (25.4%) and 78 had other genotypes (37.3%)^14^. In a Portuguese population the PiZZ phenotype was significantly associated with greater COVID incidence, followed by PiMS and PiSZ^15^. The Italian study found a higher relative risk of COVID-19 in presence of pre-existing lung diseases in subjects with severe AAT deficiency^14^ while in Portugal it was found that emphysema or bronchiectasis was not associated with increased rate of COVID-19 in this population^15^. It should be noted however, in both studies the prevalence of hospitalization due to COVID-19 was low.

The mortality from COVID-19 in our study population was low (0.7%), and the mean age at death among subjects who died due to COVID-19 was 76 years (± 10.7). Although the mortality in our cohort is slightly higher than that reported for the general Swedish population (approximately 0.3%)^22^ and the mean age at COVID-19–related death is lower than the corresponding figure for the general population (80–83 years during 2020–2023)^23^^,^ all patients who died in our study with COVID-19 as the underlying cause of death had severe co-morbidities—namely COPD and CVD—which are known to negatively impact survival during the pandemic^24,25^. These co-morbidities likely contributed substantially to the observed fatal outcomes in this population.

The Standardised Incidence Ratio (SIR) for severe COVID-19 indicated that subjects with severe AAT deficiency had a higher rate of COVID-19 hospitalisations than that expected from the general population. However, it should again be noted that the majority of subjects with severe AAT deficiency had mild or no COVID-19 infection during the pandemic, and of those who did have severe COVID-19, the majority also had a previous diagnosis of COPD, mean age was over 65 years and 64% were ever smokers, thereby increasing their risk of adverse outcomes associated with COVID-19 infection.

### Strengths and limitations

While previous studies have included small and heterogeneous cohorts with limited clinical detail, our study is the first to systematically evaluate COVID-19 outcomes in a large cohort of patients with severe AAT deficiency (PiZZ), including comprehensive clinical characterization, thus providing novel and clinically relevant insights. Another strength is the high diagnostic accuracy ensured by confirming the PiZZ phenotype through isoelectric focusing at a nationally certified laboratory. This study expands on our previous study, which relied on interviews and questionnaires to assess COVID-19 outcomes. By using the Swedish National Patient Register to verify COVID-19 diagnoses and the national death register to confirm COVID-19–related deaths, we address missing data and potential selection bias inherent in self-reported information. This approach provides a more objective and comprehensive overview of COVID-19 outcomes in individuals with severe AAT deficiency during the pandemic. The main limitation of this register-based study is that the cohort does not represent a random sample of all individuals with severe AAT deficiency in Sweden; the registry covers only about 30% of adults with the condition, while most remain undiagnosed. Moreover, national health-care registers do not reliably capture mild COVID-19, preventing detailed analysis of its relationship with AAT deficiency. Consequently, individuals with mild COVID-19 are included in the reference group for odds ratio estimates of moderate or severe disease, likely leading to an underestimation of associations. We also lacked complete vaccination data because the AATD registry is not linked to the national vaccination register. However, vaccination followed national prioritization guidelines, and individuals with severe AAT deficiency and relevant co-morbidities were offered early vaccination, reducing the likelihood of systematic differences in vaccination status. Finally, we excluded subjects whose COVID-19 events occurred outside the study period (March 2020–June 2023) to ensure comparability with national data. Although this resulted in loss of some information, it was necessary to maintain consistency with population-level outcome reporting.

### Clinical implications

This study provides new important insights into how individuals with AAT deficiency respond to emerging infections or pandemics. Early in the COVID-19 pandemic, both patients and healthcare professionals feared an increased risk of severe disease in this group. Our findings indicate that, in the absence of major comorbidities, AAT deficiency alone does not appear to confer a heightened risk of severe infection. This information may help guide risk assessment and management in future outbreaks.

## Conclusion

Most individuals with severe AAT deficiency experienced either mild COVID-19 managed in the community or no infection during the pandemic. Severe cases were frequently associated with pre-existing comorbidities, particularly COPD and CVD. These comorbidities likely contributed to the higher-than-expected COVID-19 hospitalization rates observed in this population compared with the general population.

## Data Availability

The datasets generated during and/or analysed during the current study are not publicly available due to the sensitive nature of the material collected and analysed but are available from the corresponding author on reasonable request ( [hanan.tanash@med.lu.se](mailto:hanan.tanash@med.lu.se) ).
